# Does primary brachial plexus surgery alter palliative tendon transfer surgery outcomes in children with obstetric paralysis?

**DOI:** 10.1186/1471-2474-12-74

**Published:** 2011-04-13

**Authors:** Atakan Aydın, Ahmet Biçer, Türker Özkan, Berkan Mersa, Safiye Özkan, Zeynep Hoşbay Yıldırım

**Affiliations:** 1 Istanbul University, Istanbul Faculty of Medicine, Department of Plastic, Reconstructive, and Aesthetic Surgery, Division of Hand Surgery, Capa, Istanbul; 2 IST-El Hand Surgery, Microsurgery, and Rehabilitation Group, Gaziosmanpaa Hospital Hand Surgery Department, Istanbul; 3 Istanbul University, Istanbul Faculty of Medicine, Department of Plastic, Reconstructive, and Aesthetic Surgery, Division of Hand Surgery, Physiotherapy Unit, Capa, Istanbul; 4 Van Teaching and Research Hospital, Van, Istanbul

## Abstract

**Background:**

The surgical management of obstetrical brachial plexus palsy can generally be divided into two groups; early reconstructions in which the plexus or affected nerves are addressed and late or palliative reconstructions in which the residual deformities are addressed. Tendon transfers are the mainstay of palliative surgery. Occasionally, surgeons are required to utilise already denervated and subsequently reinnervated muscles as motors. This study aimed to compare the outcomes of tendon transfers for residual shoulder dysfunction in patients who had undergone early nerve surgery to the outcomes in patients who had not.

**Methods:**

A total of 91 patients with obstetric paralysis-related shoulder abduction and external rotation deficits who underwent a modified Hoffer transfer of the latissimus dorsi/teres major to the greater tubercle of the humerus tendon between 2002 and 2009 were retrospectively analysed. The patients who had undergone neural surgery during infancy were compared to those who had not in terms of their preoperative and postoperative shoulder abduction and external rotation active ranges of motion.

**Results:**

In the early surgery groups, only the postoperative external rotation angles showed statistically significant differences (25 degrees and 75 degrees for total and upper type palsies, respectively). Within the palliative surgery-only groups, there were no significant differences between the preoperative and postoperative abduction and external rotation angles. The significant differences between the early surgery groups and the palliative surgery groups with total palsy during the preoperative period diminished postoperatively (p < 0.05 and p > 0.05, respectively) for abduction but not for external rotation. Within the upper type palsy groups, there were no significant differences between the preoperative and postoperative abduction and external rotation angles.

**Conclusions:**

In this study, it was found that in patients with total paralysis, satisfactory shoulder abduction values can be achieved with tendon transfers regardless of a previous history of neural surgery even if the preoperative values differ.

## Background

Obstetrical brachial plexus paralysis (OBPP) continues to be a challenge for developmental neurologists, physiotherapists, and hand surgeons all around the world, and its incidence is somehow increasing in developed countries. This increase has several possible explanations: an increased awareness of the disease, an increased number of centres that specialise in OBPP, an increased incidence of maternal diabetes and resultant macrosomia, and an increase in the average maternal age. Recent studies have reported an incidence of OBPP in developed countries of approximately 0.06 to 0.26%. In 70 to 90% of the cases, there is complete recovery from the disability; however, in the affected group, significant morbidity is expected [1-3]. OBPP can be classified according to the type of neural injury (neuropraxy, axonotmesis, neurotmesis, and root avulsions), the injury level (C5-C6 ± C7, which is also known as Erb's palsy, and global palsy, which involves C5-T1), and the age at presentation (OBPP in a newborn versus late presentation with functional deficits and/or deformity) [4,5]. Regarding the patients who present in infancy, the prognosis should be precisely foreseen. If complete recovery is not expected, plexus exploration should be performed as early as possible to improve the outcome. The clinical examination remains an important aspect of the evaluation in these cases because of the controversy surrounding the reliability of electrophysiological and imaging studies. However, there is also debate over the accurate interpretation of the clinical findings. Some authors suggest that the absence of biceps function at three months of age should be the threshold for early exploration, whereas others suggest that the absence of hand movements at three months and the absence of biceps functions at four months, or even later, should warrant exploration [4-9].

For the patients who present at older ages, there are several possible explanations for the disease presentation: patients with upper type (C5-C6 ± C7) or global palsy who underwent surgical intervention during infancy, patients who were followed conservatively, or patients could not receive appropriate medical care during infancy. Regardless of the background, the sequelae in later years share common features [8].

The common patterns of the shoulder problems in cases of late presentation depend on the underlying pathology. An inability to abduct and externally rotate the arm and associated glenohumeral and scapulothoracic abnormalities are among the most prominent of these problems [10,11].

The sequence of events in the shoulder following OBPP is as follows: 1, a global or localised (in the upper roots) injury to the preganglionic or postganglionic nerve root of the plexus; 2, reinnervation of the internal rotator and adductor muscles (which receive a rather rich neural support throughout the roots of the plexus) long before the abductor and external rotator muscles (the nervous supply of which is confined to the C5 and C6 roots), which causes an imbalance around the shoulder; 3, without treatment, this chronic dynamic imbalance results in a shortening of the dominant muscles, which include the pectorals, latissimus dorsi, and subscapularis; and 4, development of secondary joint and bone abnormalities, such as glenoid retroversion and hypoplasia, posterior subluxation and flattening of the humeral head, and shortening of the clavicle [10]. The timing of the intervention directly influences the functional outcome. Ideally, the muscle balance should be restored before the age of four [12]. If the abductor reinforcement is not possible using neurotisation (due to advanced age or marked atrophy of the paretic muscles), it can be achieved through tendon transfers. Tendon transfers, accompanied by the release of the contracted muscles, are still considered a through-and-through approach to restoring function and preventing fixed deformities of the shoulder [8]. In our clinic, the latissimus dorsi/teres major transfer to the greater tubercle of the humerus is considered to be the gold standard in patients older than two years of age and yields satisfactory results [13].

However, sometimes the abductor/external rotator function is restored with early nerve surgery or with conservative management, but some degree of deficit often remains. Antagonistic unmatched hyperactivity and synkinesis may worsen the functional picture even when relatively normal anatomy is restored [14].

In those patients who undergo nerve reconstruction, regardless of the preferred technique, the surgeon must use a surgically denervated/innervated muscle as a motor (latissimus dorsi or teres major) for the tendon transfer to improve shoulder abduction and external rotation [15].

Although the preoperative examination should determine whether the muscle being transferred is capable of generating sufficient muscle power before surgery (M ≥ M3+; British Medical Council (BMC) classification), during the operation, a purplish colour of the muscle or patchy fibro-fatty changes that indicate denervation may be observed, which creates uncertainty about whether an optimum result would be achieved with the transfer.

In this study, the results of tendon transfers for shoulder sequelae in preschool and early adolescent children with OBPP are compared between patients who had undergone neural surgery during infancy and those who did not. This study does not assess the success of early neural surgery for shoulder functions in OBPP cases: it assesses the effects of early surgery on late palliative surgery in cases of failure.

## Methods

### Study Design

This is a retrospective study that sought to investigate the functional results of latissimus dorsi and teres major transfers in OBPP patients who were referred to our clinic at various stages of their routine treatment during infancy or those who were referred after infancy (late referrals). The data used in this study were obtained from the hospital archives under supervision and with the permission of the Chief of the Department of Surgery (CA). A signed consent form that outlined the mode of treatment and the use of patients' imaging studies or laboratory data for scientific research was obtained from the legal guardians of all of the subjects included in this study. Additional consent forms (signed by the patients' legal guardians) had been obtained for the patients whose photographs or videos were to be published in particular. All of the patients underwent surgeries that were performed by the authors of this study. The authors confirm that the operative techniques used were scientifically proven. The authors also declare that the study complied with the World Medical Association's Declaration of Helsinki (1964) including its 6th revision (2008).

### Patients

A total of 91 OBPP patients who had undergone latissimus dorsi and teres major tendon transfer to improve shoulder function and prevent glenohumeral balance over a period of seven years (2002 to 2009) were included in this study. Patients were divided into four groups based on the extent of their lesions and previous history of brachial plexus surgery.

Group I comprised patients with total brachial plexus injury (C5 to T1) who had undergone primary brachial plexus surgery during the early years of their lives. A total of 13 patients with an average age of 5.4 years (range, 4.5 to 7.5 years) and a mean follow-up of 25 months (range, 4 to 67 months) after the secondary surgery were included in this group. All of these patients were referred to our clinic soon after birth or within three months of age. They were found to have total brachial plexus injury with hand involvement and had undergone surgery for nerve reconstruction at a mean of 6.6 months of age (range 3 to 12 months). The entire brachial plexus had been neurotised with nerve grafts using available roots, and only the accessory (nXI) nerve had been used for extraplexal neurotisation.

Group II comprised patients with upper type paralysis (or Erb's palsy that involved C5, C6 ± C7 roots) who had undergone brachial plexus exploration in infancy. A total of seven patients with an average age of 4.4 years (range, 3 to 6 years) and a mean follow-up of 44.4 months (range, 24 to 84 months) after the secondary surgery were included in this group. Five patients underwent neurolysis of the upper and middle trunk, and two patients underwent neurotisation of the upper trunk ± the middle trunk with nerve grafts during the early surgery.

The patients of groups I and II failed to experience good shoulder function as assessed at later follow up, which necessitated palliative surgery. Group III comprised patients with total brachial plexus injury who had not undergone surgery for early neural reconstruction due to late referral; 41 patients with an average age of eight years (range, 4 to 24 years) and a mean follow up of 35 months (range, 10 to 63 months) were included in this group.

Group IV comprised patients with Erb's paralysis who had not undergone surgery for early brachial plexus exploration due to late referral or conservative follow-up by our team. A total of 30 patients with an average age of 6.4 years (range, 4 to 16 years) and a mean follow-up of 36.6 months (range, 14 to 72 months) were included in this group. For the patients in groups III and IV, the preoperative examinations and direct roentgenograms revealed a congruent glenohumeral joint but less than satisfactory shoulder abduction and external rotation that necessitated palliative surgery.

The distribution of the patients among the groups are shown in Table 1.

**Table 1:** Groups and distribution of patients among groups

Previous nerve surgery	Injury Type	Group	Number of Patients	
Yes	Total	I	13	20
	Erb's	II	7	

No	Total	III	41	71
	Erb's	IV	30	

### Evaluation of Patients

All patients were consecutively evaluated by the physiotherapy team and the surgical team. Active and passive ranges of motion were measured with goniometric video-graphic analyses by the physiotherapist and/or the surgeons. Videographic analyses were captured with Windows Media Player 11^® ^(Microsoft Corporation 1 Microsoft Way, Redmond, WA, USA) and assessed using ImageJ 1.34 s software (public domain of the National Institutes of Health, Maryland, USA).

### Surgical Technique

A modified Hoffer technique [16] was used for improving shoulder abduction and external rotation. Patients were positioned in the lateral decubitus position. The entire affected extremity was cleaned, prepped, and draped in standard sterile fashion to allow full mobility during the operation; this was important because manoeuvres were frequently utilised intraoperatively to assess joint mobility and ease the transfer. The conjoint tendon of the latissimus dorsi and teres major were palpated along the axillary border, and their locations were marked. A posterior zigzag incision was made along the lateral scapular border to avoid skin contractures. The conjoint tendon and the muscles were extensively dissected while carefully preserving their neurovascular pedicles to provide full excursion. Following the detachment of the tendon from the inner medial humerus, a tunnel was created between the long head of the triceps muscle and deltoid muscles, which is the location of the axillary nerve. The tissue was carefully handled even if the nerve had little or no motor ability; even in those cases, the axillary nerve still typically contained a substantial number of afferent fibres. A curved incision was made over the belly of the deltoid muscle to access the greater tubercle of the humerus, where the supraspinatus, infraspinatus and teres minor muscles attach. Immediately below the insertion of the infraspinatus muscle, a suture anchor was applied directly into the bone. This incision and the prepared tunnel were unified, and the suture material (2/0 polyester) was passed posteriorly towards the first incision. The conjoint tendon was woven with the suture, and together they were passed anteriorly through the tunnel back to the second incision. Before the completion of the transfer, passive range of motion was checked, and if it was found to be suboptimal, the tightness of the pectoralis major muscle and the subscapularis muscle were assessed by palpation. If necessary, the pectoralis tendon was fractionally lengthened to undermine the first incision anteriorly. The subscapularis muscle was released from the anterior surface of the scapula (the authors currently prefer to use botulinum toxin A for subscapularis hyperactivity rather than extending the release because this muscle is an important stabiliser of the shoulder joint [4]). The suture and the tendon were secured to the reinsertion site while the arm was positioned in 90° of abduction and in external rotation. A custom-made splint, which was prepared preoperatively, was applied either in the operating room (if reliable cooperation was established with the family and the child was able to cooperate) or six weeks later following cast immobilisation. Physical therapy was started six weeks after the operation.

### Statistical Analyses

Preoperative and postoperative mean abduction and external rotation values were analysed to determine any statistically significant differences between groups I and II, groups III and IV, groups I and III, and groups II and IV, separately. The one way ANOVA (analysis of variance) method was used with SPSS version 14.0 (SPSS Inc., 233 S. Wacker Drive, 11th floor, Chicago, Illinois 60606). A p value of 0.05 determined significance.

## Results

**Figure 1:**
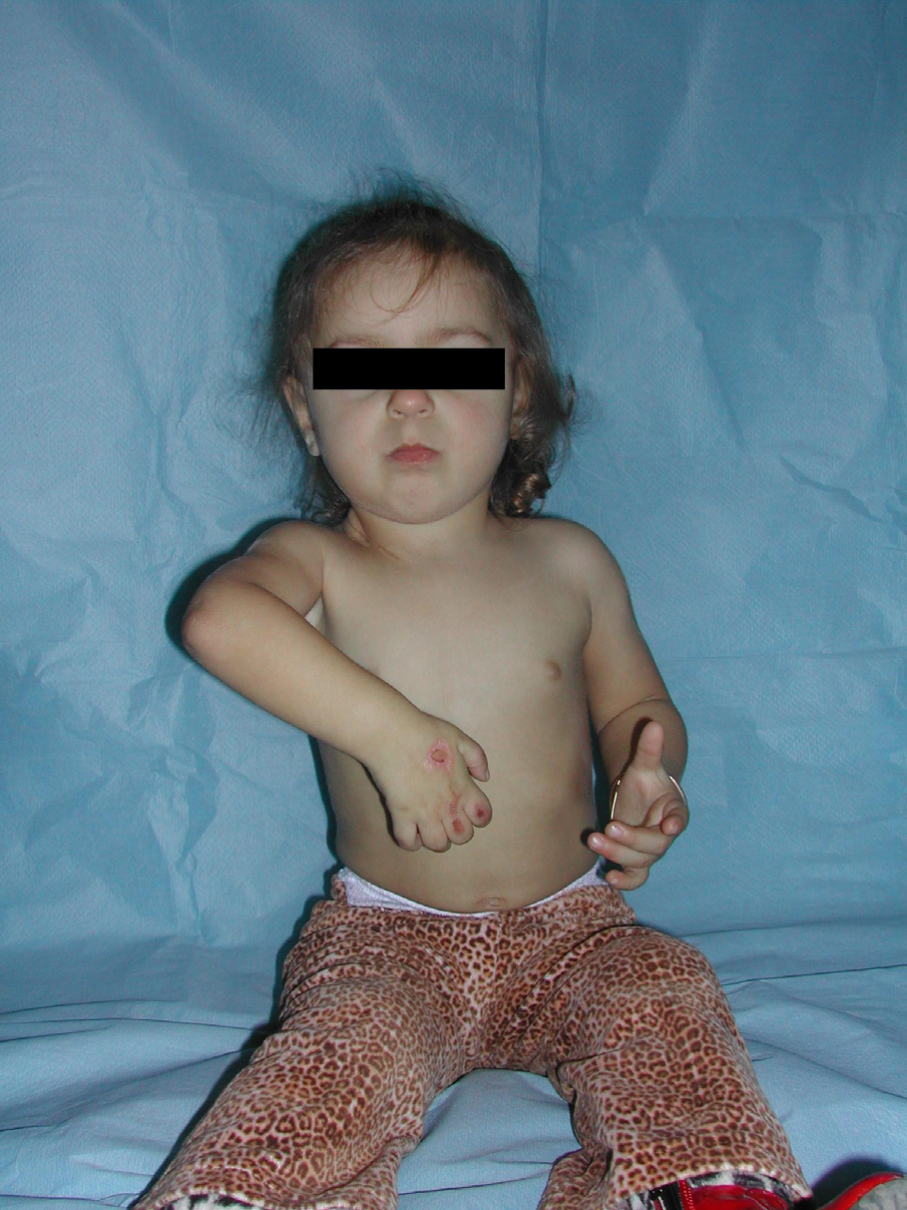
**A patient with total paralysis who underwent brachial plexus neurotisation in infancy**. The neural surgery failed to improve the patient's shoulder function to a satisfactory level. The videos of the patient before brachial plexus exploration can be seen in Additional Files 1 and 2.

**Figure 2:**
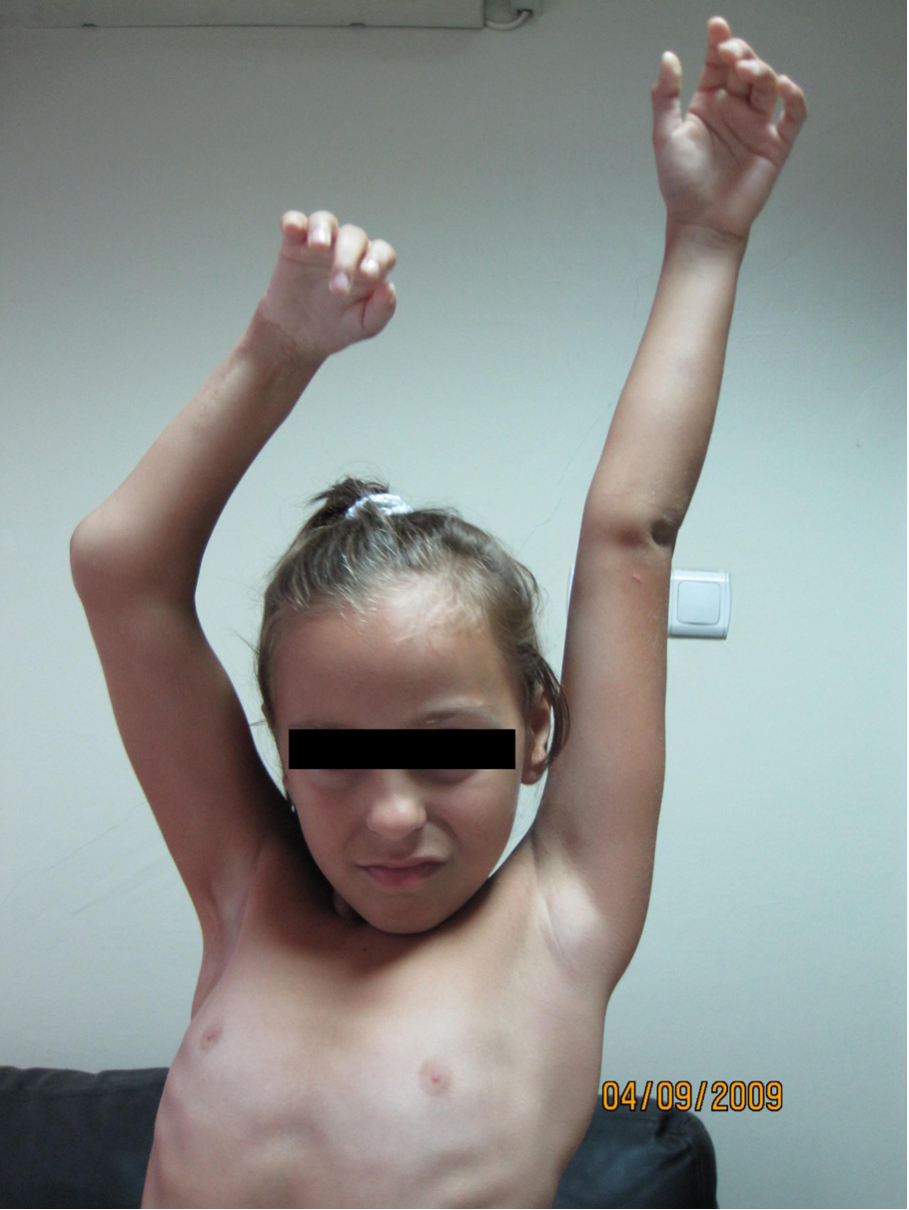
**Successful transfer of the latissimus dorsi and teres major conjoint tendon yielded adequate abduction of the patient's arm**.

**Figure 3:**
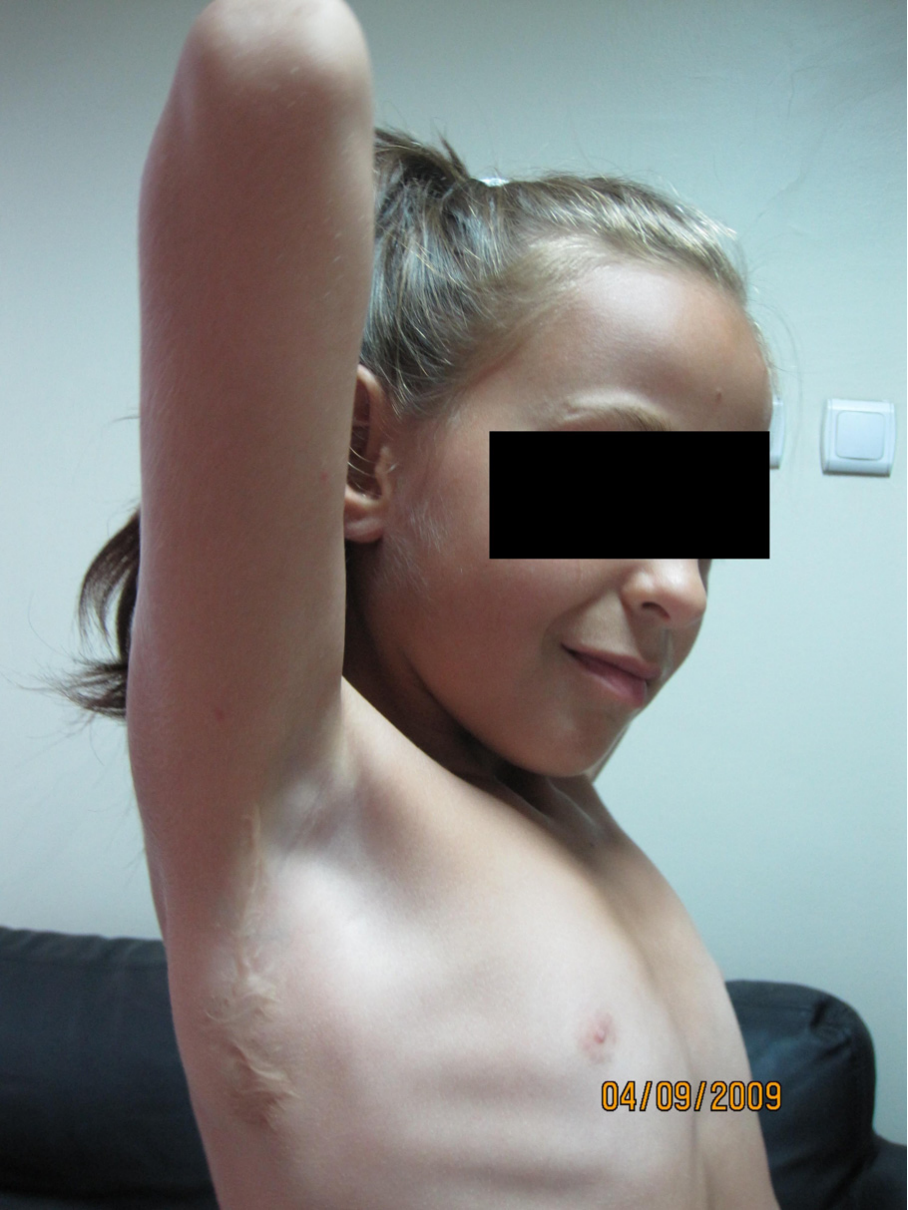
**External rotation could have been established by the same transfer**.

**Figure 4:**
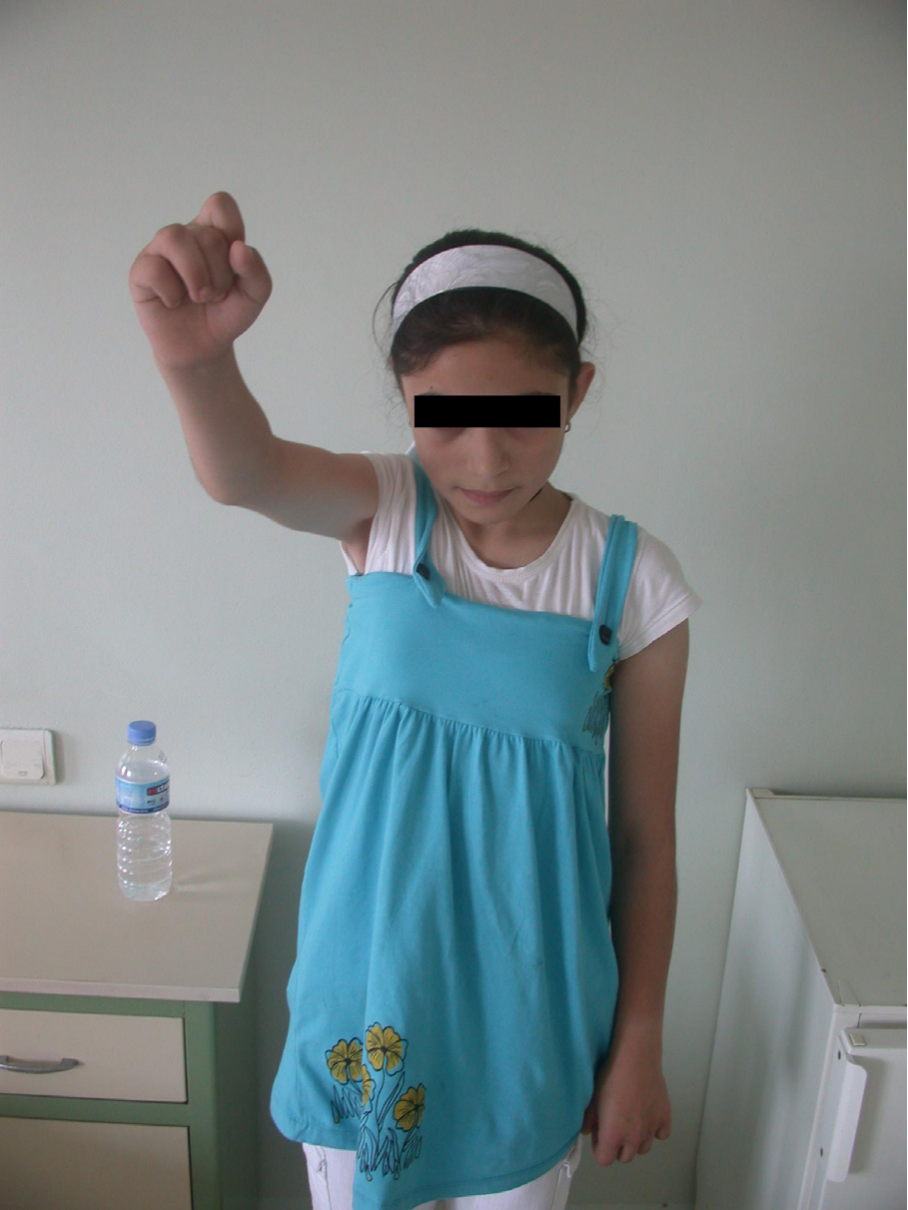
**A patient with Erb's paralysis who presented at a later age (six years old) with disabilities around the shoulder**. Shoulder abduction before transfer is shown.

**Figure 5:**
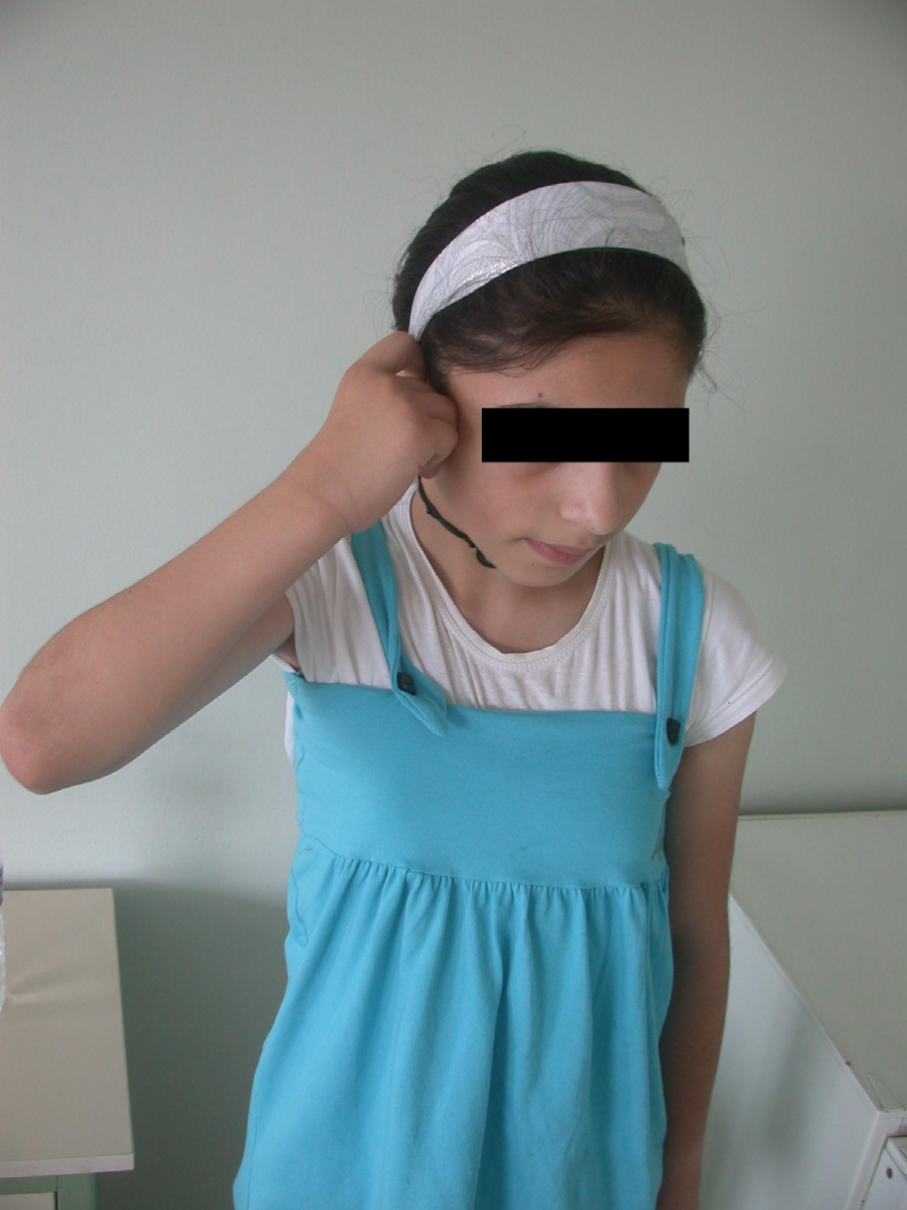
**Preoperative view of the same patient attempting to externally rotate her arm**.

**Figure 6:**
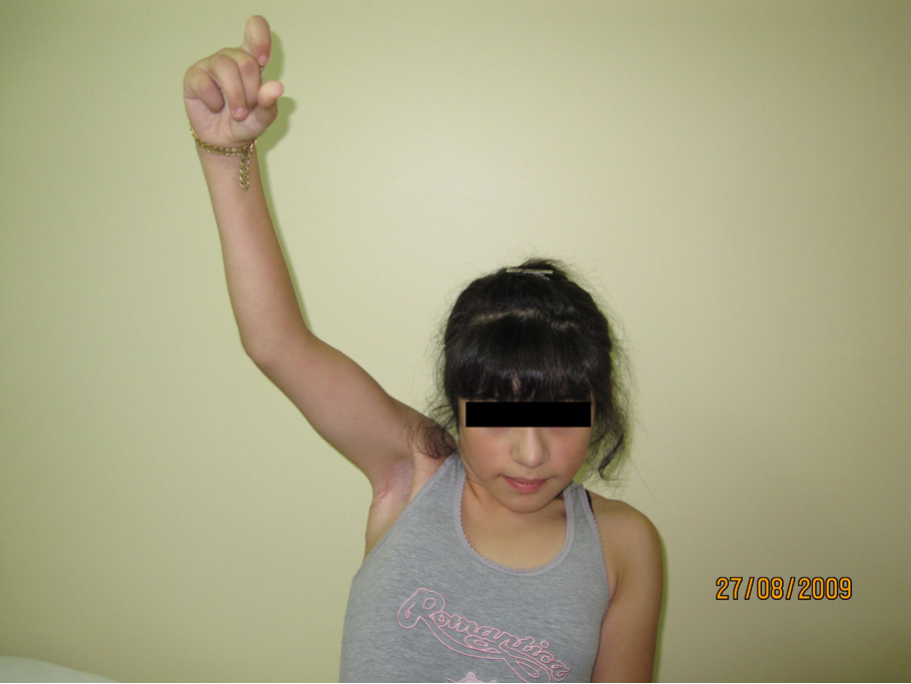
**Shoulder abduction of the patient with late presentation following the transfer**.

**Figure 7:**
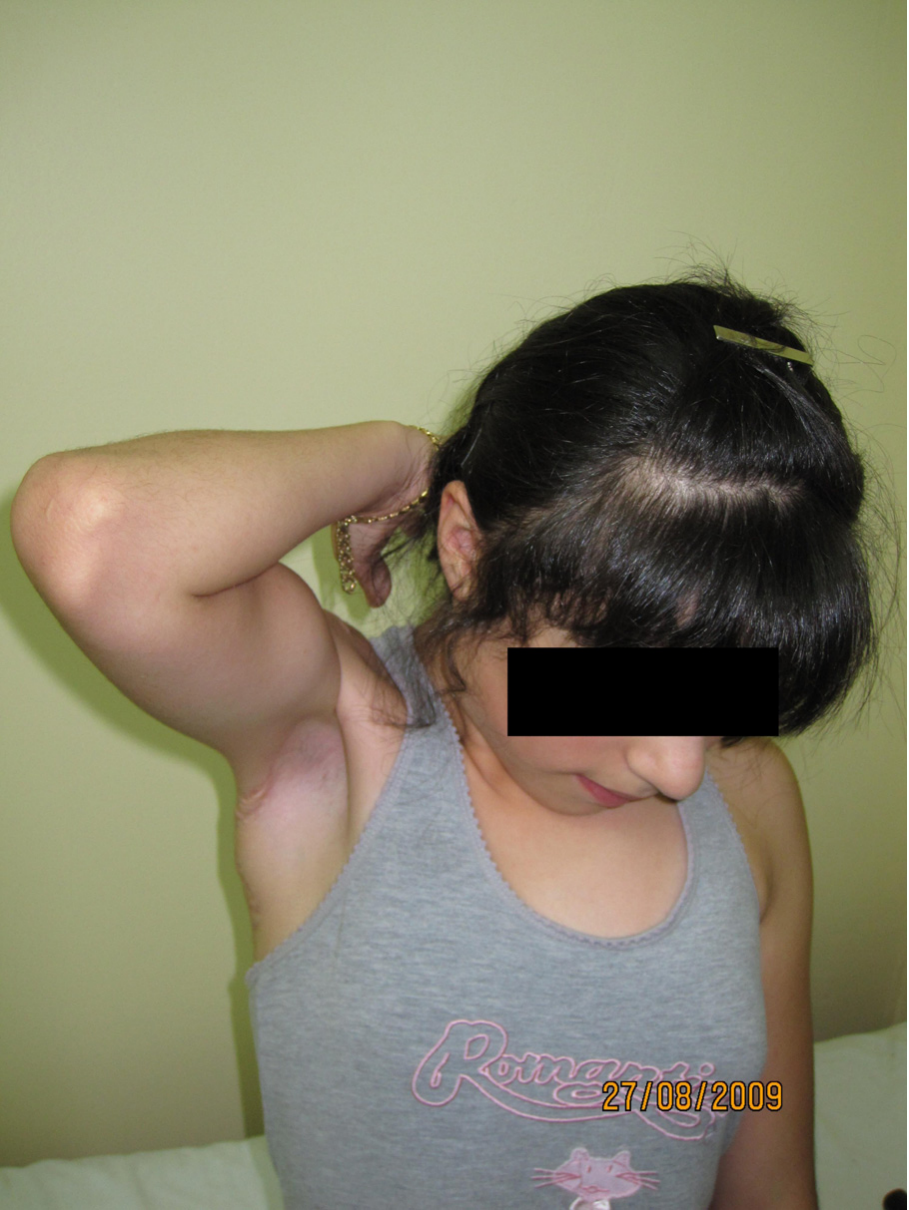
**External rotation of the patient with late presentation following the transfer**.

In groups I, II, III, and IV, the mean abduction values increased by 70°, 71.5°, 58°, and 63°, respectively; whereas in groups I, II, III, and IV, the mean external rotation was increased by 8.1°, 48°, 45°, and 45°, respectively (Table 2). Table 3 summarises the results of the measurements and the demographic characteristics of the groups. Preoperative and postoperative patient photos and videos are shown in Figures 1, 2, 3, 4, 5, 6, and 7, and videos of the patients who underwent neural surgery can be found in Additional Files 1 and 2. Within the early surgery groups (I and II), only the postoperative external rotation angles were statistically larger (F = 4.57, p < 0.05) in the Erb's palsy patients. Within the palliative surgery-only groups (III and IV), there were no significant differences between the preoperative and postoperative abduction and external rotation angles. Within the groups of patients with total palsy (groups I and III), the patients who had not undergone neural surgery in infancy showed significantly larger preoperative abduction values (F = 8.97, p < 0.05). However, the postoperative comparison yielded no significant differences (F = 2.53, p > 0.05). The preoperative external rotation values yielded no significant differences (F = 2.51, p > 0.05), whereas group III showed a significant postoperative increase (F = 15.4, p < 0.05). Within the upper palsy groups (II and IV), there were no significant differences between the preoperative and postoperative abduction and external rotation angles.

**Table 2:** Net gain in abduction and external rotation for the separate groups

	Group I	Group II	Group III	Group IV
Abduction Gain (°)	70	71.5	58	63
External Rotation Gain(°)	8.1	48	45	45

**Table 3:** Demographic information, preoperative and postoperative findings

	Early Nerve Surgery at Infancy Groups (N: 20)				No Previous Surgery Groups (N:71)			
	Group I Preop	Group I Postop	Group II Preop	Group II Postop	Group III Preop	Group III Postop	Group IV Preop	Group IV Postop
Number of Patients	13		7		41		30	
Mean Age* at Operation	5.4		4,4		8		6.4	
Mean Follow-up**	25		44.4		35		36.6	
Mean Abduction***	45	115	49.2	120.7	75	133	74	137
External rotation***	16.9	25	22	70	30	75	27	72

*: Mean age, years. **: Follow-up period, months.***: Active ranges of motion in terms of degrees.

## Discussion

The first attempts at nerve reunion for obstetrical brachial plexus lesions occurred at the very beginning of the last century [17]. Although the fundamental surgical approach has not changed significantly, the incorporation of microsurgery in the field has undoubtedly improved the outcomes over the last three decades.

Reinforcement of the paretic, if not flaccid, abductors/external rotators is achieved by intraplexal/extraplexal neurotisations within the first two years of life. If there is a usable available C5 root, neurotisation can be accomplished via a short graft to the post-injury upper trunk, to the posterior division of the upper trunk, or directly via a longer graft to the suprascapular nerve and axillary nerve [18]. For the patients with global obstetrical paralysis, total avulsions are extremely rare, in contrast to traumatic brachial plexus lesions. Rupture of the upper roots with avulsion of the lower roots at their extremes commonly occurs in patients with traumatic brachial plexus lesions. In these cases, neurotisation of the cords with available upper roots may be performed. However, the suprascapular nerve should be supported with extraplexal donor nerve tissue (most commonly, the accessory nerve). However, C5/C6 avulsions are not uncommon with breech presentations. Various types of nerve transfers are performed to supply the suprascapular and axillary nerves outside of the plexus. The sources of these nerves include the platysmal branch of the facial nerve, the spinal accessory nerve, the hypoglossal nerve, the phrenic nerve, the deep cervical motor nerves, the intercostal nerves, and the contralateral C7. However, most of these transfers are hypothetical. The accessory nerve is most commonly used for suprascapular neurotisation due to its anatomic availability, fascicular capacity, and limited morbidity [19,20]. Nerve reconstructions are clearly indicated for children with total (C5-T1) involvement. However, for upper type (C5, C6 +/- C7) injuries, the absolute indication for surgical exploration is still unclear. With his unmatched experience with obstetrical brachial plexus palsy and its surgical treatment, Gilbert proposed the absence of biceps function at three months of age as the threshold for surgical exploration and repair [6]. Many authors acknowledge the importance of early surgery after various wait-and-see periods [6,7,14,21-24]. Electrophysiological studies typically rely on human factors, and a high level of cooperation between the surgical and the neurological teams is required. Imaging studies have not yet been shown to produce reliable and definitive outcomes. Recent publications suggest limitations for surgical indications in cases with co-contractions (which may present as an inability to flex the elbow and abduct and externally rotate the shoulder although some contractions in the affected muscles may also be observed). These studies reported excellent results with botulinum toxin A combined with appropriate physiotherapy in those cases with co-contraction-related functional disabilities [14,25,26]. Some authors also described results of worsening of glenohumeral deformity in cases in which surgery was performed early for upper type palsies [27,28]. A better understanding of the co-contraction mechanisms between the shoulder abductors and adductors as well as the elbow flexors and extensors led some surgeons to re-evaluate their decisions about early nerve surgery based on elbow flexion or shoulder abduction. Some authors preferred late (1-4 years) distal neurotisations (Oberlin's partial ulnar to musculocutaneous transfer) if the child was capable of sufficient shoulder abduction but insufficient elbow flexion [29,30].

Our clinical algorithm has evolved over the years; we reserve nerve reconstruction for established total palsy patients (at three months of age, a thorough clinical evaluation, including electrophysiological studies, is conducted) with hand involvement. Botulinum toxin A is extensively used for upper type obstetrical palsies if a clinical or electrophysiological basis for co-contractions develops. If some degree of disability persists beyond 12 months, the Oberlin transfer is preferred for elbow flexion, and the shoulder abduction/external rotation deficits are subsequently addressed with tendon transfer. Our experience with the modified Hoffer technique is quite satisfactory for the muscular imbalance and the shoulder disability [13]. In this study, the effects of a previous history of neural surgery on the outcomes of later transfers, such as the Hoffer, were investigated. In this study, the major observation may have been that among the patients in our clinic who were scheduled for latissimus dorsi/teres major to tuberculum majus transfer, the preoperative active shoulder abduction differed significantly between groups I and III only (the two groups with total palsy: group I with previous history of surgery and group III without previous history of surgery). However, this inconsistency disappeared following the operation. This finding seems to be important because the latissimus dorsi muscle with its denervated appearance still works adequately as a shoulder abductor following the transfer. Before such a tendon transfer operation, it is typically confirmed that the muscle being transferred has at least M3 (British Medical Council) muscle strength; a history of denervation and reinnervation as well as the perioperative appearance of the muscle cast doubt about the possible outcomes in some cases. To our knowledge, such a study has not yet been published in the current literature. Another important finding of this study was that in the group with total paralysis and previous history of neural surgery (group I), the postoperative ranges of motion in terms of shoulder external rotation were significantly lower compared to both the upper palsy with a previous history of surgery group (group II) and the total palsy without a previous history of surgery group (group III). One possible reason for this finding may be the state of the glenohumeral joint. Magnetic resonance studies are required to confirm this theory, and this imaging modality is not routinely performed in our clinic. Passive ranges of motion are always assessed before surgery and therefore should have yielded satisfactory ranges; however the postoperative gains (only 8.1° as compared to 45° and 48° for the other groups) were significantly less than expected. As mentioned above, Nath and Liu found that the glenohumeral deformity is worsened if the patient had a previous history of neural surgery [28], and a glenohumeral deformity may affect the external rotation more significantly than abduction. This may be evidence for the aforementioned study. However, in group II, the group with Erb's paralysis and previous history of neural surgery, both the preoperative and postoperative values were comparable to the palliative groups; therefore, additional studies that investigate the exact effects of neural surgery on the joint and the distinction between the impact of surgery and birth on the plexus and the joint are required.

## Conclusions

The results of this study indicate that late reconstructions using a "reinnervated" muscle yield outcomes that are comparable to those obtained by utilising previously untouched muscles. If the need for palliative surgery around the shoulder emerges during the course of obstetrical brachial paralysis treatment, the latissimus dorsi transfer option should still be considered, even for patients with a previous history of primary neural surgery. There is no "missed opportunity" for late reconstructions for patients with upper type of birth palsies even if they underwent early nerve reconstruction operations.

### Limitations of the study

The study was retrospective, which produces some limitations, such as a decreased level of confidence and reduced case optimisation. The cases that were included in this study required treatment to address the shoulder function limitations. The reasons for the failure of the primary surgery (if one was performed) or its frequency were beyond the scope of this study. These aspects of the current study obviate the need for future prospective studies on this subject.

## Competing interests

There are no financial or ethical conflicts of interest associated with this study. The study was funded in part by the research commission at our university and therefore by the government of the Turkish Republic.

## Authors' contributions

TÖ, AA and AB contributed to the study design, the final draft of the manuscript and performed the operations. SÖ and ZHY contributed to the preoperative and postoperative evaluation of the patients and conducted the physiotherapy following the operations. All of the authors read and approved the final manuscript.

## Pre-publication history

The pre-publication history for this paper can be accessed here:

http://www.biomedcentral.com/1471-2474/12/74/prepub

## Supplementary Material

Additional file 1 **Video 1. **Preoperative assessment of an infant with obstetric paralysis, part 1. Click here for file

Additional file 2 **Video 2. **Preoperative assessment of an infant with obstetric paralysis, part 2. Click here for file
